# En bloc multivisceral resection for a giant retroperitoneal dedifferentiated liposarcoma: technical considerations

**DOI:** 10.1093/jscr/rjag982

**Published:** 2026-10-27

**Authors:** Sadik Al-Hassani, Akinfemi Akingboye, Ilenia Merlini, Salomone Di Saverio

**Affiliations:** Department of General Surgery, The Dudley Group NHS Foundation Trust, Pensnett Road, Dudley, West Midlands, DY1 2HQ, United Kingdom; Department of General Surgery, The Dudley Group NHS Foundation Trust, Pensnett Road, Dudley, West Midlands, DY1 2HQ, United Kingdom; College of Health and Life Science, Aston Medical School, School of Medicine, Pharmacy and Biosciences, Aston University, Birmingham B4 7ET, UK, United Kingdom; Department of Gastrointestinal Surgery AST Ascoli Piceno, Madonna del Soccorso Hospital, San Benedetto del Tronto, Italy; Department of Gastrointestinal Surgery AST Ascoli Piceno, Madonna del Soccorso Hospital, San Benedetto del Tronto, Italy

**Keywords:** liposarcoma, retroperitoneal neoplasms, multivisceral resection, pancreatectomy, nephrectomy

## Abstract

Retroperitoneal sarcomas are rare, aggressive malignancies that often present late owing to the retroperitoneum’s anatomical expansibility. Dedifferentiated liposarcoma is the most common high-grade subtype. We describe the technical management of a 65-year-old man with a giant retroperitoneal dedifferentiated liposarcoma involving multiple adjacent organs, treated by radical en bloc multivisceral resection including the kidney, adrenal gland, spleen, and distal pancreas. Postoperative recovery was complicated by a Grade B pancreatic leak and a left pleural effusion, managed with percutaneous and thoracic drainage, respectively. Histology confirmed a dedifferentiated liposarcoma resected with clear (R0) margins. At 18 months, no radiological recurrence had been observed, although this follow-up interval remains short relative to the recurrence interval reported for high-grade dedifferentiated liposarcoma. This case illustrates the technical conduct of multivisceral resection for a giant retroperitoneal sarcoma and the perioperative surveillance required to manage expected complications such as pancreatic leak.

## Introduction

The retroperitoneum is an expandable space that contains several retroperitoneal organs (kidneys, adrenal glands, part of the duodenum), major abdominal vessels, abdominal lymphatics, and sympathetic lumbar chains [1]. Approximately 80% of retroperitoneal tumours are malignant [2]. The majority of malignant retroperitoneal tumours are soft tissue sarcomas (STS) [2]. STS are aggressive mesenchymal neoplasms, with an estimated incidence of 4–5/100 000 per year in Europe [2]. Approximately 10%–15% of all adult STS are located retroperitoneally [1].

Retroperitoneal sarcomas typically occur between the ages of 50 and 60, affecting men and women equally [1]. Radical surgical excision is the primary treatment; achieving R0 has been shown to improve cure rates and reduce local recurrence [2]. However, multivisceral resection is necessitated in cases of local infiltrations [2].

We report the technical considerations of laparotomy and en bloc multivisceral resection in a 65-year-old man with a large retroperitoneal sarcoma.

## Case presentation

A 65-year-old male presented with left-sided back pain radiating to the left leg, initially attributed to sciatica. He was on antihypertensive medication only. Examination revealed a large, palpable, non-tender, left-sided mass, and mild anaemia (Hb 10.8 g/dl).

An initial magnetic resonance imaging (MRI) scan of the spine (performed for the patient’s sciatica) revealed a large, hyperintense retroperitoneal mass situated between the left kidney and the left psoas muscle, which appeared infiltrated. Subsequent computed tomography (CT) imaging demonstrated a similar mass measuring ~20 × 12 × 12 cm, compressing the diaphragm cranially (Fig. 1A) and displacing the spleen and left kidney anteriorly (Fig. 1B). The mass displayed extensive infiltration into adjacent structures, including the large para-aortic nodes, left psoas muscle, lateroconal fascia, Gerota’s fascia, posterior renal fascia, perirenal fat, and left hemidiaphragm. A preoperative CT-guided biopsy performed with an 18-gauge needle was reported as showing leiomyosarcoma. Staging CT of the thorax showed no metastatic disease.

**Figure 1:**
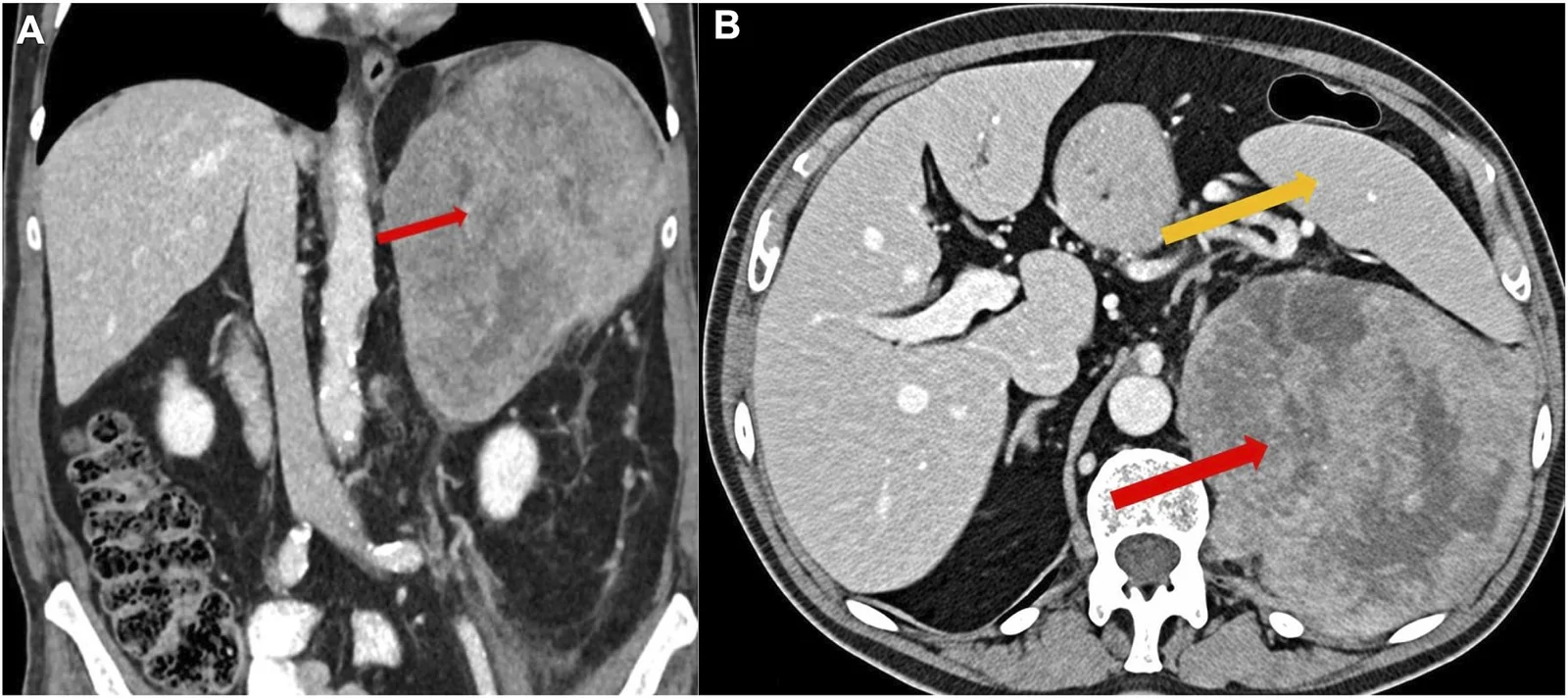
(A) Coronal view of CT scan showing retroperitoneal mass (red arrow) compressing the diaphragm cranially; (B) axial view of CT scan showing retroperitoneal mass (red arrow) displacing the spleen (orange arrow).

Pre-operative assessment showed a World Health Organisation (WHO) performance status of 2 and an American Society of Anaesthesiologist grade (ASA grade) of 3, reflecting the patient’s tumour burden and hypertension. The multidisciplinary team advised operative management.

### Surgical technique

A midline xiphopubic laparotomy with left lateral and subcostal extension was performed. Left colonic mobilization was carried out, with the line of Toldt incised and the left and transverse colon mobilized medially to expose Gerota’s fascia, which formed the roof of the tumour compartment. The left colon itself showed no macroscopic tumour deposit and was therefore carefully dissected free rather than resected en bloc, avoiding the added risk of a bowel anastomosis in an already high-risk operation.

The spleen was removed separately, and not as part of the colonic mobilization, to improve access to the retroperitoneum and posterior diaphragm. This also achieved oncological clearance of the tumour-infiltrated lateroconal fascia, which was removed en bloc with the splenectomy specimen; the splenectomy itself was not the technical focus of the procedure. Anteriorly, the gastrocolic and gastrosplenic ligaments, tethered to the mass, were partially divided using a stapler, with division of the short gastric vessels.

Posteriorly, the body and tail of the pancreas were mobilized by tunnelling beneath the gland, lateral to the splenomesenteric–portal confluence. The splenic artery and vein were ligated individually. The pancreas was transected at the isthmus using a stapler, and the stump, including the main pancreatic duct (duct of Wirsung), was reinforced with continuous 3–0 Prolene sutures.

The tumour infiltrated the posterior diaphragmatic pillars and left hemidiaphragm, necessitating en bloc resection of the posterior diaphragm, exposing the thoracic cavity and left lung (Fig. 2A), which appeared unaffected. Two large 30 Fr thoracic drains were inserted (Fig. 2B), and the diaphragmatic defect was closed with double-layer continuous 0-Vicryl sutures, reinforced with a pedicled omental fat patch. Prosthetic mesh reinforcement was not used, given the high risk of mesh infection in the presence of intra-abdominal contamination and collections.

**Figure 2:**
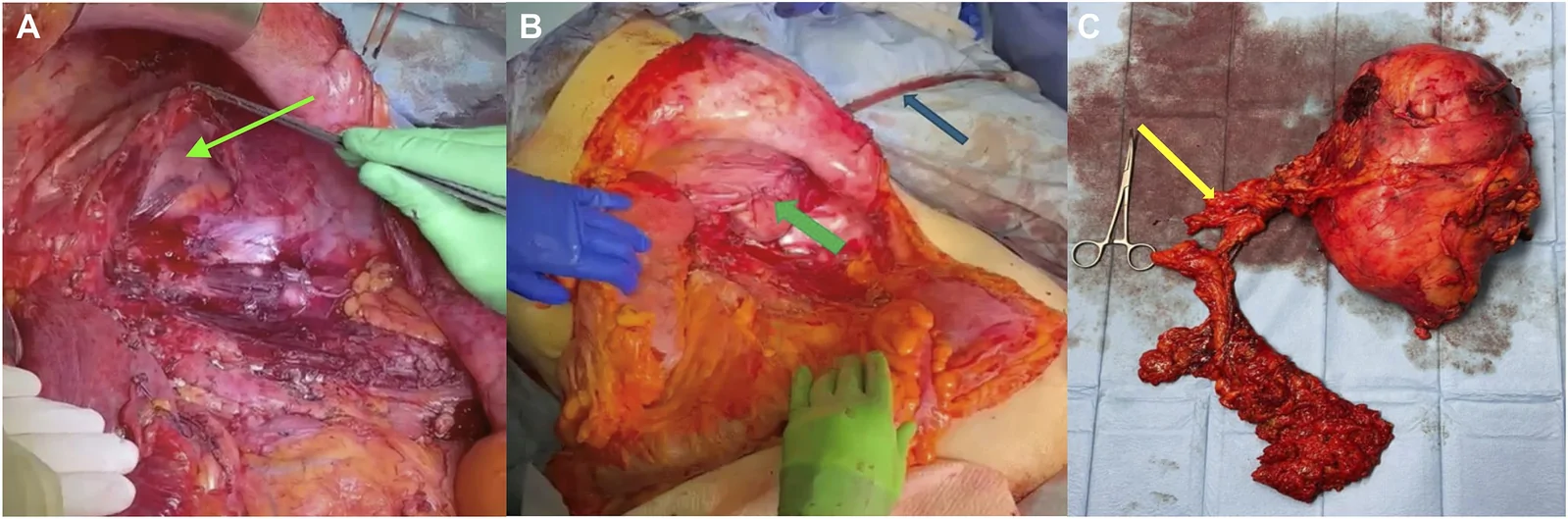
(A) Intraoperative image showing excised diaphragm with exposed lung underneath (green arrow); (B) intraoperative image showing diaphragm repair (green arrow) and drain at the base of the left lung (blue arrow); (C) excised sarcoma en bloc specimen with left kidney and ureter (yellow arrow).

Tumour resection extended to the abdominal wall musculature, paravertebral muscles, dorsolumbar vertebrae, and left lateral abdominal wall soft tissues. Due to infiltration of the posterior renal fascia and perirenal fat, a left nephroureterectomy was performed. The level of ureteric division was performed distally. The renal vein was ligated at its inferior vena cava confluence, and two renal arteries were ligated at their aortic origin using Hem-o-Lok clips and 0-Vicryl sutures. The anatomical variant of two left renal arteries was identified on preoperative CT imaging.

The proximal and mid iliopsoas muscles were excised, preserving only the distal portion. A peri-aortic and inferior mesenteric artery lymphadenectomy was also performed. Although this is not routine practice in retroperitoneal sarcoma, the enlarged lymph nodes identified on preoperative CT imaging could not be disregarded; these were dissected individually, not en bloc with the specimen. The ligament of Treitz was detached to complete the retroperitoneal dissection, and a left adrenalectomy was included. The final specimen is shown in Fig. 2C. Estimated blood loss was 1500 ml, and three units of red blood cells and four units of fresh frozen plasma were transfused intraoperatively. Total operative time was 9 h and 44 min.

### Outcome and follow-up

The patient was managed in the intensive care unit postoperatively.

Postoperative recovery was complicated by two events, graded Clavien–Dindo IIIa overall. First, a Grade B pancreatic leak, per the International Study Group of Pancreatic Surgery (ISGPS) 2016 classification, presented with postoperative fever and negative blood cultures; the patient remained otherwise asymptomatic and was treated empirically with broad-spectrum antibiotics and percutaneous drainage. Second, a small iatrogenic lung parenchymal laceration with associated pneumothorax was managed with a chest drain for ~13 days.

CT at 2 weeks demonstrated a left pleural effusion and a left subphrenic (abdominal) collection (Fig. 3A and B). The pleural effusion was managed by thoracentesis; fluid analysis was serosanguineous with no malignant cells identified. The abdominal collection corresponded to the pancreatic leak described above (elevated drain amylase) and was drained percutaneously. Neither collection required further intervention; both resolved on interval CT surveillance at 8 weeks.

**Figure 3:**
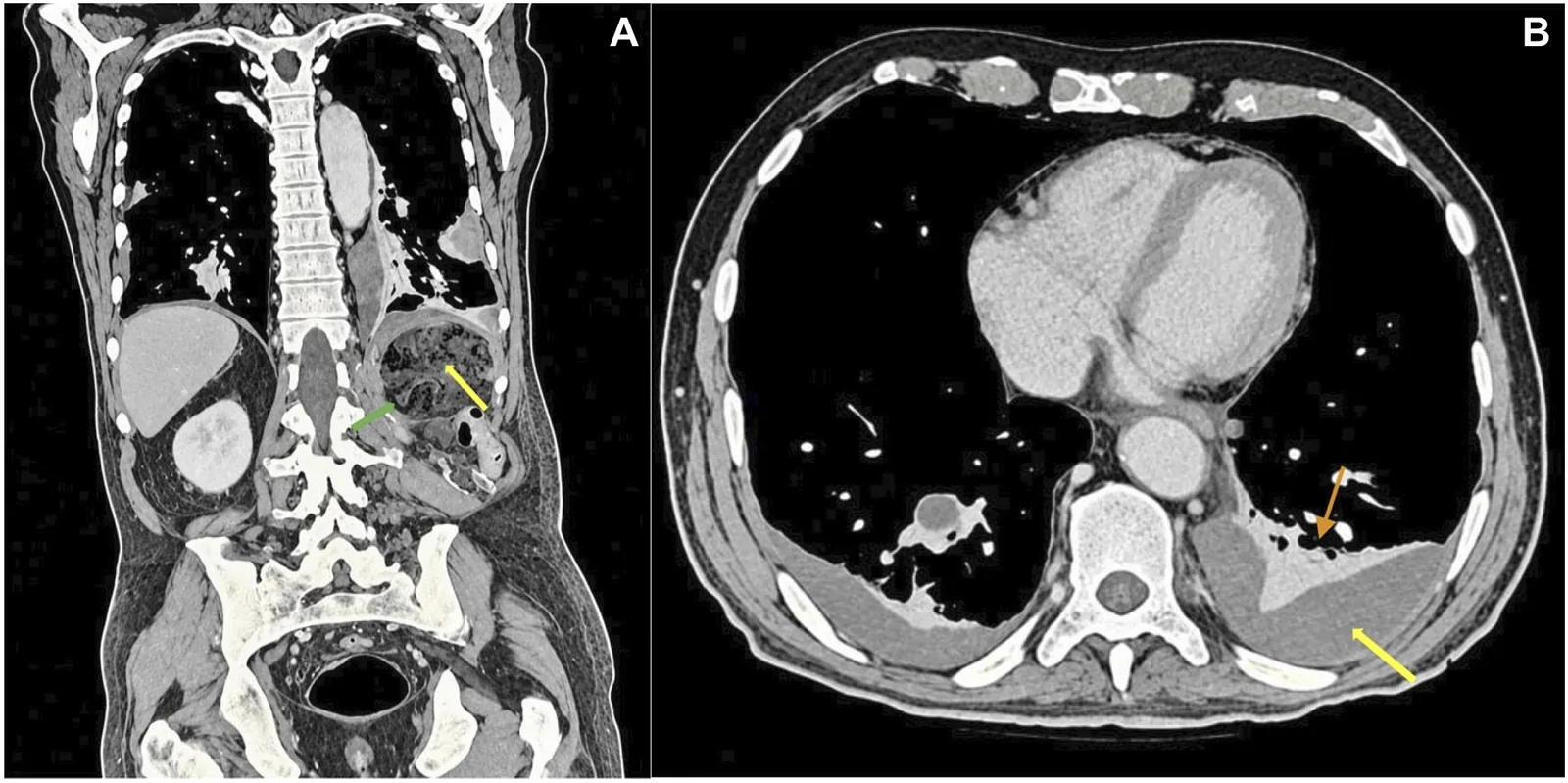
(A) Coronal view of CT scan showing the abdominal cavity following removal of the dedifferentiated liposarcoma (green arrow) and the left subdiaphragmatic collection (yellow arrow); (B) axial view of CT scan showing left subdiaphragmatic collection (yellow arrow) and left-sided pleural effusion (orange arrow).

Renal function was serially monitored following left nephroureterectomy. Pre-operative baseline values were creatinine 0.8 mg/dl and urea 34 mg/dl. These rose during the intensive care stay to creatinine 1.5 mg/dl and urea 83 mg/dl and peaked on the ward at creatinine 1.9 mg/dl and urea 120 mg/dl, before improving to creatinine 1.2 mg/dl and urea 64 mg/dl prior to discharge. At outpatient follow-up, renal function had returned towards baseline, with creatinine 0.9 mg/dl and urea 37 mg/dl at the first check and creatinine 0.9 mg/dl and urea 30 mg/dl at the second check, reflecting adequate compensatory function of the contralateral kidney. The patient’s total length of stay was 39 days. Figure 4 shows a clinical timeline with the patient’s renal function at each stage.

**Figure 4:**
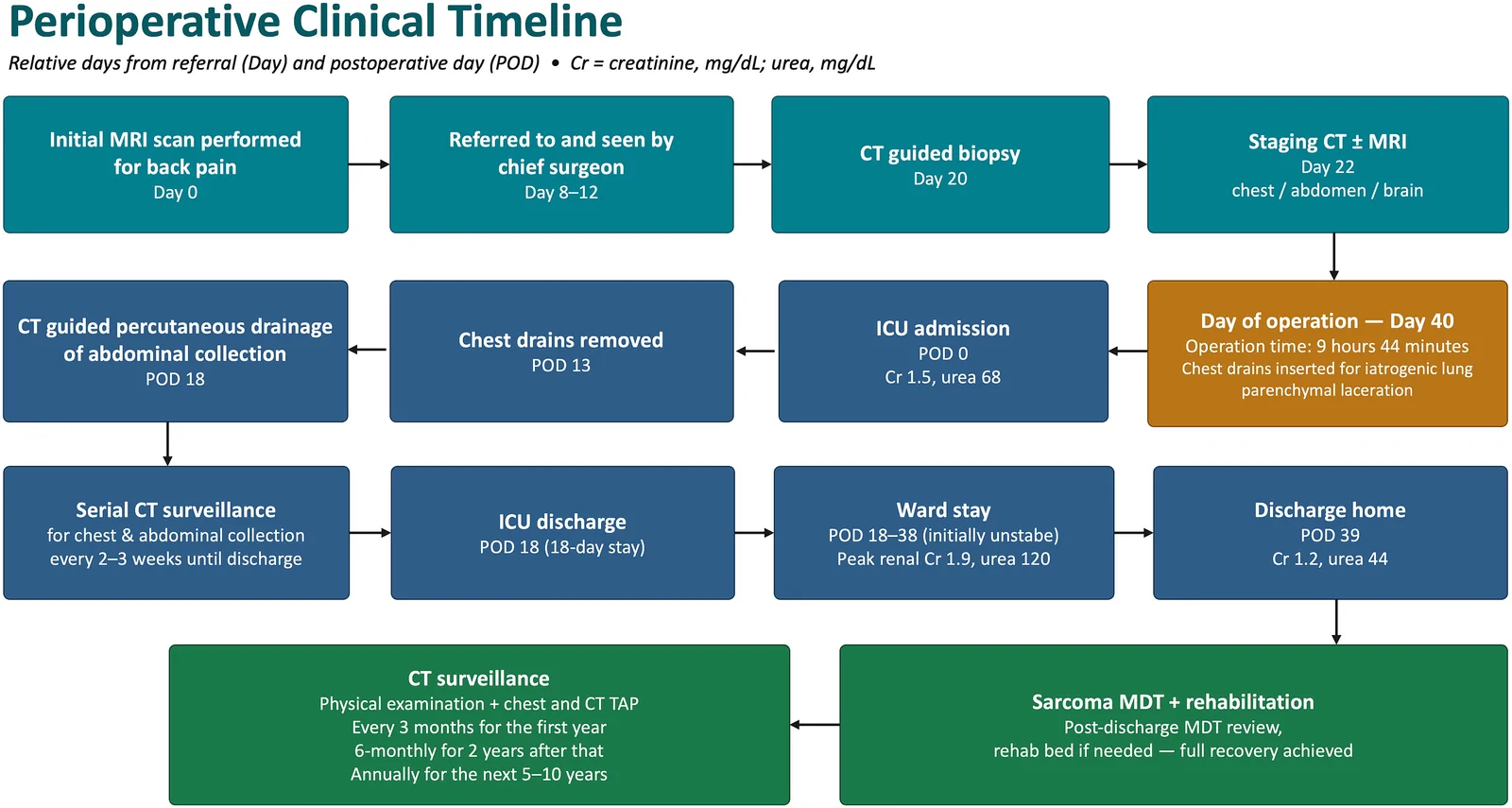
Perioperative clinical timeline: explaining patient's journey from the time of referral to full recovery and the subsequent surveillance period.

During the septic episode associated with the pancreatic leak, the patient received broad-spectrum antimicrobial cover with daptomycin, meropenem, cefiderocol, and tigecycline. Post-splenectomy antimicrobial and vaccination prophylaxis were instituted according to unit protocol. Vaccination prophylaxis comprised conjugated pneumococcal vaccine, meningococcal ACWY a (conjugate vaccine that protects against four strains of Neisseria meningitidis bacteria: groups A, C, W, and Y) and meningococcal B vaccines, *Haemophilus influenzae* type b vaccine, and seasonal influenza vaccination, to be repeated annually.

Surgery followed ~40 days after initial imaging, during which interval radiological growth occurred; the resected specimen measured 30 × 25 cm and weighed 3.8 kg. Histopathology confirmed a French Federation of Cancer Centers Sarcoma Group (FNCLCC) grade 3 retroperitoneal dedifferentiated liposarcoma with myogenic differentiation, with a clearance margin of >1 mm but <10 mm (R0). A total of nine lymph nodes were examined (five para-aortic, four lumbar), all free of metastatic involvement, as was the psoas muscle margin. Immunohistochemistry, including Mouse Double Minute 2 homolog (MDM2), alpha-smooth muscle actin, caldesmon, and desmin, supported the diagnosis. The oncology team advised that, given the radicality of resection and the clear margins achieved, further molecular subtyping was unlikely to alter management, and surgical follow-up alone was recommended.

Following iliopsoas resection, the femoral, obturator, and genitofemoral nerves were preserved. Hip flexion and quadriceps function were preserved postoperatively; the patient had mobility weakness on the affected side, which improved with regular physiotherapy, with full functional recovery achieved by 8 weeks. No major sensory or motor deficit was present at 18-month follow-up.

Surveillance comprised CT imaging at 3-monthly intervals for the first 6 months, 6-monthly for the following 2 years, and yearly thereafter for 3 years, in keeping with general sarcoma surveillance principles. At 18-month follow-up, no radiological recurrence, diaphragmatic hernia, or eventration was identified; however, this interval remains short relative to the recurrence dynamics of high-grade dedifferentiated liposarcoma, where local recurrence commonly occurs beyond 2 years, and longer surveillance is required before durable local control can be concluded.

## Discussion

Most malignant retroperitoneal tumours are STS [2], comprising liposarcoma (50%), leiomyosarcoma, and other rarer types. According to the WHO Classification of Tumours of Soft Tissue and Bone, subtypes include well-differentiated, dedifferentiated (aggressive and high-grade), myxoid, and pleomorphic variants [3].

MRI is the preferred imaging modality for STS; however, in retroperitoneal sarcomas, CT is equally effective for diagnosis [4]. CT allows for assessment of fat planes, organ involvement, and distinction between well-differentiated and dedifferentiated components, which aids surgical planning [4]. The European Society of Medical Oncology (ESMO) recommends en bloc resection with any adherent organs as standard treatment for retroperitoneal sarcomas [4]. The goal is to achieve R0 or R1 margins, as local recurrence and tumour grade are key prognostic factors [1]. Multivisceral resection is associated with high mortality, particularly when the pancreas or duodenum is involved [2]. In this case, multivisceral resection was safely performed, and residual collections were surgically drained and monitored with serial CT imaging.

Poor outcomes are associated with incomplete resection and intraoperative tumour rupture [1, 5]. Minimally invasive approaches may be suitable for smaller and well-differentiated tumours, but an open approach remains the standard when multivisceral resection is indicated. The Surgery with or without Preoperative Radiotherapy in Retroperitoneal Anadigmatic Soft Tissue Sarcoma (STRASS) trial (Bonvalot *et al*.) [6] found that preoperative radiotherapy did not improve overall abdominal recurrence-free survival in retroperitoneal sarcoma, with subgroup analyses suggesting effects may differ by histological subtype and grade; on this basis, ESMO guidance does not mandate radiotherapy for dedifferentiated liposarcoma [4]. Table 1 summarizes reported cases of giant retroperitoneal sarcoma managed with aggressive en bloc resection between 2015 and 2025; this is a narrative rather than a systematic selection of cases, and a formal systematic search strategy was not undertaken, which we consider beyond the scope of this technical note. Two patients received adjuvant chemotherapy (Table 1) [5, 7].

**Table 1:** Reported cases of giant retroperitoneal sarcoma managed with en bloc resection (2015–25).

Citation (reference number)	Year	Patient demographic	Neoadjuvant therapy	Approach	Organs resected	Margin	Adjuvant therapy	Histology	Tumour size/weight	Follow-up/recurrence
Hassani *et al*. [5]	2025	65, M	None	Laparotomy, en bloc resection	Kidney	R1	Chemotherapy (not specified)	Dedifferentiated liposarcoma	40 × 27 × 17 cm Weight NR	Death after 1 year due to chemotherapy-related complications
Vlăsceanu *et al*. [7]	2023	54, M	None	Laparotomy, en bloc resection	Inguinal ligament and right testis	Not stated	Chemotherapy with doxorubicin, ifosfomide, mesna	Myxoid liposarcoma	11.7 × 13.1 × 20.3 cm Weight NR	6 cycles of chemotherapy. No recurrence in follow-up at time of publication
Herrera-Almario *et al*. [8]	2022	53, M	None	Laparotomy, en bloc resection	None	Not stated	None	Well-differentiated liposarcoma	70 × 90 × 100 cm Weight 26 kg	No recurrence reported. Length of follow-up NR
Almas *et al*. [9]	2020	43, F	None	Laparotomy, en bloc resection, Hartmann’s	Kidney, colon	R0	None	Well-differentiated liposarcoma	50 × 40 × 30 cm Weight NR	No recurrence at 1 year
Zhang *et al*. [10]	2015	48, F	None	Laparotomy and left partial nephrectomy	Kidney	Not stated	None	Myxoid liposarcoma	30 × 20 × 15 cm Weight NR	Recurrence at 3 months

NR = not reported in the source available to us.

In our case, radical multivisceral resection achieved R0, with no radiological recurrence at 18 months; however, this interval remains insufficient to conclude durable local control. Reported outcomes for comparable cases vary widely. Vlăsceanu *et al*. [7] achieved improved local control after multivisceral resection of a myxoid liposarcoma, though their patient remained on adjuvant chemotherapy with follow-up still ongoing. Zhang *et al*. [10] reported recurrence within 3 months despite aggressive resection of a similarly sized myxoid liposarcoma, highlighting that tumour biology and margin status, rather than resection extent alone, drive local control. These cases suggest that R0 resection does not guarantee durable control, particularly for higher-grade or myxoid subtypes, and that our own 18-month outcome warrants longer surveillance before being considered representative.

This case was managed outside a designated sarcoma referral centre, although the operating surgeon has extensive experience in sarcoma surgery within a high-volume unit. ESMO and consensus guidance recommend centralization of retroperitoneal sarcoma care, and this should be considered when interpreting our outcomes. With careful case selection, radical excision with multivisceral resection remains a feasible technical approach to achieving R0 resection in large, aggressive retroperitoneal tumours, ideally within a dedicated sarcoma surgical unit.
